# The Associations of Physical Activity and Health-Risk Behaviors toward Depressive Symptoms among College Students: Gender and Obesity Disparities

**DOI:** 10.3390/ijerph21040401

**Published:** 2024-03-26

**Authors:** Samantha Moss, Xiaoxia Zhang, Ziyad Ben Taleb, Xiangli Gu

**Affiliations:** 1 Kinesiology Department, State University of New York at Cortland, Cortland, NY 13045, USA; samantha.moss02@cortland.edu; 2 School of Sport Sciences, West Virginia University, Morgantown, WV 26506, USA; xiaoxia.zhang@mail.wvu.edu; 3 Department of Kinesiology, University of Texas at Arlington, Arlington, TX 76019, USA; ziyad.bentaleb@uta.edu

**Keywords:** depression, physical activity, health-risk behaviors, young adults, tobacco

## Abstract

Engaging in health-risk behaviors (HRBs) may be correlated with depressive symptoms among college students, but these relationships require more research. The purpose of this study was to examine the associations of physical activity levels (i.e., light [LPA] and moderate–vigorous [MVPA]) and HRBs (i.e., sedentary behavior [screen-based and non-screen-based behavior] and cigarette and e-cigarette tobacco use) with depressive symptoms in a sample of college students. Physical activity levels and HRBs were assessed through validated questionnaires. In total, 366 students participated (M_age_ = 22.59 ± 3.54; 60.1% female; 52.9% normal weight). E-cigarette use in males (β = 0.23, *p* < 0.05) and screen-based sedentary behavior in females (β = 0.14, *p* < 0.05) showed significant predictive utility toward depressive symptoms. In the overweight/obese group, screen-based sedentary behaviors (β = 0.19, *p* < 0.05) and e-cigarette use (β = 0.23, *p* < 0.01) showed significant predictive utility toward depressive symptoms. Females reported higher levels of depressive symptoms (M_female_ = 18.23 vs. M_male_ = 14.81; η^2^ = 0.03) and less MVPA (M_male_ = 52.83 vs. M_female_ = 41.09; η^2^ = 0.06) than males. Enhancing mental health by improving physical activity and eliminating HRBs should be tailored toward at-risk demographics.

## 1. Introduction

Depression is one of the most common mental disorders in the United States [1]. It can adversely influence one’s academic performance and increase the risk of disability, premature mortality, and suicide [2,3,4]. People in their early adulthood (18–25 years old) have the highest prevalence of depression (17% in 2020) compared to all other age groups [1]. Young adults, specifically those entering college/university, experience important transitions, developments, and lifestyle changes (e.g., enrolling in universities, staying away from family members, and facing financial challenges), which increase their vulnerability to mental health issues such as depression [5,6]. These existing statistics call for immediate action to address depression among college-aged individuals.

Moreover, early adulthood is also an influential time in which health and/or health-risk behaviors (HRBs) may persist and continue throughout the lifespan [7]. According to the Centers for Disease Control and Prevention, HRBs are defined into four major components for adults (≥18 years), namely binge drinking, lack of sleep, smoking, and physical inactivity [8]. Specifically, HRBs such as physical inactivity and cigarette or e-cigarette use have been documented to cause harmful health consequences such as obesity and cardiovascular diseases [9,10,11,12]. Even with the known health benefits of physical activity participation and cessation of smoking tobacco products, nearly half of adults do not meet the 150 min/week guideline of physical activity and 15.5% of adults are reported smokers [13,14,15].

Within the past 12 years, new evidence has emerged to identify sedentary behavior as an HRB, independent from time spent in physical activity [16]. Similar but not synonymous with physical inactivity, sedentary behavior refers to activities that are completed, or the time spent, in a seated or lying position expending low amounts of energy [17]. These activities can be further dichotomized into screen-based and non-screen-based behaviors; that is, activities involving multimedia screen usage would be allotted to time spent in screen-based behavior, while other activities not using multimedia screens would be allotted to non-screen-based behaviors. A recent systematic review indicated that computer use was reported as the most frequented modality of screen-based behaviors in adults [18]. Excessive sedentary behaviors, especially screen-based behaviors, have been found to increase the risk of cardiometabolic diseases and all-cause mortality [16,19]. While the impact of sedentary behaviors on health has been well investigated, the combined effects of sedentary behaviors and other HRBs (e.g., smoking) are less researched and require greater attention.

Lubans and colleagues [20] proposed a conceptual model that outlines the effects of physical activity on mental health outcomes such as cognitive function, well-being (e.g., quality of life, global self-esteem, etc.), and ill-being (e.g., anxiety, depression, etc.) through three different mechanisms. Specifically, it has been suggested that physical activity affects an individual’s mental health status through neurobiological, psychosocial, and behavioral pathways. In terms of the behavioral mechanism, it has been hypothesized that individuals’ other behaviors such as sleep and sedentary behaviors could interact with physical activity and have combined effects on their mental health status. This conceptual model also suggests that the patterns of physical activity (e.g., frequency, intensity, time, type, and context) may moderate the proposed effects. Indeed, research has reported significant associations between depression and physical inactivity, sleep, and sedentary behavior among college-aged young adults [21,22,23]. For example, grounded by Lubans’ conceptual model, Zhang and colleagues [24] found significant mediatory effects of sedentary behaviors and sleep on physical fitness and depression among female college freshmen. Another recent systematic review concluded that screen-based sedentary behavior was associated with a higher risk of depression, especially when it exceeded 2 h/day [25]. Using this theoretical concept as a foundation provides comprehensive theory-guided interpretations.

Along with the previously highlighted HRBs of physical inactivity and excess sedentary behavior, college students are extremely susceptible to engaging in tobacco product use such as e-cigarettes [26]. Accordingly, tobacco use has been reported to be significantly associated with adverse mental health effects such as depression and anxiety [27,28,29]. However, few studies have simultaneously investigated the effects of HRBs (e.g., physical inactivity, excess sedentary behavior, and tobacco use) on mental health and whether they can contextualize lifestyle behaviors of college-aged young adults, which will provide insight into more advanced and comprehensive health promotional strategies.

It is well known in the current literature that, from adolescence through adulthood, females are twice as likely as males to be diagnosed with depression and exhibit depressive symptoms [30]. There are also strong indications of positive associations between mental health status and weight status, but further research is needed to understand their relationships [31]. The current study adds a unique contribution to the literature by exploring how these various associations of HRBs with mental health may differ between males and females, as well as between healthy weight and overweight/obese young adults. Thus, guided by the theoretical framework established by Lubans and colleagues [20] the purpose of this study was to examine the associations of different physical activity levels (e.g., light physical activity and moderate–vigorous physical activity) and HRBs (sedentary behavior [screen-based and non-screen-based behavior] and cigarette and e-cigarette tobacco use) with depressive symptoms in college students. Gender and obesity disparities in physical activity levels and HRBs were also investigated in the sample.

## 2. Materials and Methods

### 2.1. Participants

A total of 366 college students (60.1% female; mean age = 22.59 years, SD = 3.54) were recruited online in the summer and fall of 2020. All participants were from one public university in North Texas. More than half of these participants (59.6%) majored in Kinesiology, 36.3% majored in Nursing, and 4.1% were from other majors. The university’s institutional review board approved this study (Protocol #2019-0027). Participants signed an online consent form prior to answering the survey.

### 2.2. Procedures

All the data collection procedures were carried out online. The survey, including the informed consent form and questionnaire, was created using the QuestionPro web-based survey software (QuestionPro Inc., Austin, TX, USA). The approved recruiting email and electronic flyer were sent out to recruit participants mainly through course instructors, personal contacts, and snowball sampling. Participants voluntarily filled out the survey and submitted it online through the QuestionPro system.

### 2.3. Depressive Symptoms

The 20-item Center of Epidemiologic Studies Depression Scale (CES-D) [32] was used to assess participants’ depressive symptoms. Participants reported the frequency of a symptom they had within the past 7 days from rarely/less than 1 day (0), a little/1–2 days (1), occasionally/3–4 days (2), to always/5–7 days (3). Sample questions include “I was bothered by things that usually don’t bother me” and “My sleep was restless”. The total depression score was summed from the 20 items (ranging from 0 to 60), where a higher score indicates greater/severe depressive symptoms. Cronbach’s α showed sufficient internal consistency of this scale (α = 0.75).

### 2.4. Physical Activity Behaviors

The Godin Leisure-Time Physical Activity Scale was used to assess different intensities of physical activity [33,34]. Participants recalled the frequency of engaging in light (e.g., yoga golf), moderate (e.g., fast walking, baseball, etc.), and vigorous (e.g., running, strenuous long-distance cycling, etc.) activities in the last 7 days. The weight score index (LSI) was obtained using the LSI formula for light PA (frequency of light × 3) and moderate–vigorous physical activity (MVPA; frequency of moderate × 5 + frequency of vigorous × 9). This scale demonstrated to have good test–retest reliability, with coefficients ranging from 0.70 to 0.91 among college students [35].

### 2.5. Health-Risk Behaviors

#### 2.5.1. Sedentary Behaviors

The screen-based and non-screen-based sedentary behaviors were measured by the self-report Sedentary Behavior Questionnaire (SBQ) for adults [36]. Four items assessed screen-based behaviors, namely watching television, using the computer for study, using the computer for entertainment, and sitting while talking or texting on the phone. Another four items assessed non-screen-based sedentary behaviors, namely sitting while doing non-computer schoolwork, doing paperwork not related to school, reading and creating art, and driving/taking a car or bus. Participants reported how much time they spent per day in a typical week on those eight sitting activities. The sum of the time from the four activities in screen-based and non-screen-based sedentary behaviors, respectively, was used in the final data analysis. The internal consistency of the scale was acceptable (Cronbach’s α = 0.61).

#### 2.5.2. Cigarette and e-Cigarette Use

Participants responded to two questions assessing their usage of cigarettes and e-cigarettes. Participants were asked “What best described your cigarette use during the past month (30 days)?”, and four responses were available such as “did not use cigarettes in the past month”, “used cigarettes less than once a week”, “used cigarettes at least once a week but not every day”, and “used cigarettes every day or almost every day”. The same question format was used to assess the usage of e-cigarettes. Participants’ responses were further coded into 0, 1, 2, and 3. The average score from the question was used in the data analysis representing the participants’ use of cigarettes and the use of e-cigarettes.

### 2.6. Data Analysis

The data analysis was carried out using SPSS 29.0 (IBM Corp, Armonk, NY, USA). All participants provided sufficient data in the survey, and less than 2% of missing values were detected. Little’s MCAR (missing completely at random) test was applied to test patterns of missing data, and the results indicated a completely missing at random (chi-square = 9.00, df = 9, *p* = 0.44); thus, no data imputation was processed [37]. Subsequently, four sequential steps of data analysis were conducted. First, descriptive statistics (mean and standard deviation) were calculated for study variables (e.g., depressive symptoms; physical activity behaviors: MVPA and light physical activity; HRBs: screen-based sedentary behavior, non-screen-based sedentary behavior, the use of cigarettes, and the use of e-cigarettes). Secondly, Pearson product–moment correlation analysis was used to examine the binary associations among study variables. Thirdly, the hierarchical multiple regression was conducted to determine the role of physical activity behaviors (Step 1) and HRBs (Step 2) on depressive symptoms in the healthy weight group and the overweight/obese group, respectively, after accounting for gender (Step 1). The R square and standardized beta coefficient (β) were reported. Finally, we examined the disparity of gender (male vs. female) and weight status (healthy weight group vs. overweight/obese group) in study variables. In particular, the analysis of variance (ANOVA) model on depressive symptoms, a multivariate analysis of variance (MANOVA) model on physical activity behaviors, and a MANOVA model on HRBs were performed. Partial eta-square (η^2^) was used to detect the effect sizes (small = 0.01; moderate = 0.06; and large = 0.1) [38]. An alpha level of 0.05 in a two-tailed test was set up for statistical significance.

## 3. Results

### 3.1. Sample and Demographic Characteristics

Overall, a total of 366 college students (60.1% female) were included in the data analysis. Half of them (51.9%) were healthy weight, and 29% and 17.8% of them were overweight and obese, respectively. The participants were from diverse racial groups, with 30.9% of White, 23.2% of Hispanic, 16.1% of Black, 10.1% of Asian, and 19.7% of other races or mixed races. Nearly half of the participants were senior college students (44.8%), followed by 22.7% being juniors, 17.8% being graduate students, 7.9% being sophomores, and 6.8% being freshmen (Table 1).

### 3.2. Correlations among Variables

The results of Pearson’s product–moment correlation analysis are presented in Table 2. MVPA was significantly and negatively associated with depressive symptoms (r = −0.12; *p* < 0.05), while screen-based sedentary behaviors (r = 0.17; *p* < 0.01) and the use of e-cigarettes (r = 0.14; *p* < 0.01) were positively associated with depressive symptoms. There was a significant, negative association between MVPA and screen-based sedentary behaviors (r = −0.12; *p* < 0.05). Light physical activity showed a small but significant association with the use of e-cigarettes (r = −11; *p* < 0.05).

### 3.3. Predictive Utilityof Behaviors on Depressive Symptoms

Table 3 presents the results of the two-step hierarchical regression analyses in predicting depressive symptoms among males and females, respectively. Among males, the model accounted for 12% of the variance in depressive symptoms, where e-cigarette use served as the only significant predictor (β = 0.23, *p* < 0.05). Among females, the model accounted for 7% of the variance in depressive symptoms; however, only screen-based sedentary behavior was the marginally significant predictor (β = 0.14, *p* = 0.05).

Table 4 presents the results of the two-step hierarchical regression analyses in predicting depressive symptoms in the healthy weight group and the overweight/obese group, respectively. In the overweight/obese group, the model was significant and accounted for 14% of the variance in depressive symptoms. Both screen-based sedentary behaviors (β = 0.19, *p* < 0.05) and the use of e-cigarettes (β = 0.23, *p* < 0.01) were significant predictors of depressive symptoms. In other words, overweight and obese young adults who had more screen-based sedentary behaviors and more frequent use of e-cigarettes were more likely to have greater depressive symptoms after controlling for their gender and physical activity behaviors. The model of the normal weight group was insignificant, and neither the physical activity behaviors nor the HRBs significantly contributed to depressive symptoms.

### 3.4. Gender and Weight Status Disparities

The ANOVA results pointed to the significant main effect of gender on depressive symptoms (F_1,355_ = 10.15, *p* < 0.01, η^2^ = 0.03). There was no main effect for weight status nor an interaction effect between gender and weight status. Females reported significantly higher depressive symptoms than their male counterparts (M_female_ = 18.23 vs. M_male_ = 14.81; η^2^ = 0.03). The MANOVA model on physical activity behaviors yielded a significant effect of gender (Wilks’ Lambda = 0.94; F_1,355_ = 10.15, *p* < 0.001, η^2^ = 0.06). Males engaged in significantly more MVPA than females (M_male_ = 52.83 vs. M_female_ = 41.09; η^2^ = 0.06). Neither a main effect for weight status nor an interaction effect between gender and weight status was observed on physical activity behaviors. The MANOVA results of HRBs revealed a marginally significant interaction effect of gender and weight status (Wilks’ Lambda = 0.97; F_4,349_ = 4.00, *p* = 0.05, η^2^ = 0.03) but no main effects of either gender or weight status. A further univariate analysis showed that females with a healthy weight had significantly more screen-based sedentary behaviors than their male peers with a healthy weight (M_female_ = 10.14 vs. M_male_ = 7.97; η^2^ = 0.02). The participants had similar patterns of using cigarettes and e-cigarettes regardless of gender and weight status. These disparities are presented in Table 5.

## 4. Discussion

Implementing a theoretical-based approach, this study comprehensively examined the associations of multiple levels of physical activity (light physical activity and moderate–vigorous physical activity) and HRBs (screen-based and non-screen-based behaviors and cigarette and e-cigarette use) with depressive symptoms among college-aged young adults and uncovered gender and obesity disparities among this group. The results highlighted strong associations between HRBs and young adults’ depressive symptoms. In particular, our findings revealed that e-cigarette use in males, screen-based sedentary behavior in females, both e-cigarette use and screen-based sedentary behavior in females, and both e-cigarette use and screen-based sedentary behavior in overweight/obese young adults were significantly associated with depressive symptoms. These gender and obesity disparities are not only identified but can also be used for insightful intervention strategies that are specifically tailored to individuals most at risk (e.g., female young adults).

It is important to note the significant correlations of depressive symptoms with MVPA and HRBs (screen-based sedentary behavior and e-cigarette use); that is, individuals with less MVPA and more screen-based sedentary behavior and e-cigarette use are at a greater risk of having depressive symptoms. Aligned with current research, other studies have also reported inverse associations between MVPA and mental health issues in young adults [38,39,40,41]. Moreover, the association between screen-based sedentary behavior and depression has also been observed in existing studies [21]. Furthermore, current findings agree with previous studies that e-cigarette use is significantly related to depressive symptoms [29,42]. This finding provides evidence that e-cigarette users may lack the ability to cope with stressful events (e.g., communication skills, social support, etc.) and therefore will rely on e-cigarette use as a coping mechanism, which only provides a transitory relief and no long-term mental health benefits [43,44].

These findings support the need to adopt strategies that are specifically tailored to reduce health-risk behaviors such as promoting MVPA, reducing screen-based behaviors, and/or preventing e-cigarette use among the young adult population so as to potentially reduce the risk of depression. Moreover, the overall findings highlighted the distinct gender effect on the relationship between HRBs and depressive symptoms. Specifically, e-cigarette usage played a significant role in developing depressive symptoms in males but not in females regardless of physical activity and other HRBs examined in this study. This is an important finding as e-cigarettes are more commonly used by males than by females [45]. Previous research showed mixed findings regarding the relationship between e-cigarette use and depressive symptoms [46]. This finding adds critical evidence to the literature indicating that the association between e-cigarette use and depressive symptoms may be stronger among males than among females. Curbing e-cigarette use through education and cessation strategies not only has the potential to eradicate physical health consequences but can also address mental health issues among young adult males. As for females, screen-based sedentary behavior demonstrated a significant role in explaining the potential risk of depressive symptoms. The findings also showed that females were more inactive and exhibited higher depressive symptom scores than their male counterparts. These findings suggest that reducing screen-based behaviors (e.g., mobile phone usage, social media engagement, etc.) may be more beneficial for relieving depressive symptoms among college-aged females. Even though young adults are required to use computers and screens more frequently than in the past, taking breaks during screen-based behaviors may be a feasible way to negate the potentially harmful mental health issues that could arise.

In addition, we also found that individuals who had more HRBs were more likely to show higher depressive symptoms, and this association differed between healthy and overweight/obese individuals. Specifically, screen-based sedentary behavior and e-cigarette use showed combined effects on depressive symptoms in the overweight/obese group. These findings highlight the vulnerability of overweight/obese young adults and the potential mental health risks due to their increased participation in HRBs. Previous research has demonstrated a strong connection between obesity and cigarette smoking, and more recently, this relationship has been extended to e-cigarette usage as well [47]. For instance, one recent study investigated some HRBs (i.e., smoking, nutrition, sleep, etc.) in European college students and found that smoking was a significant predictor of BMI and self-related health perception using regression analyses [48]. The research literature noted that the overweight and obese rate among young adults in the US remains elevated at 39.8% [49]; in the same vein, this study reported that almost half of the sample was categorized as overweight/obese. Given the high prevalence of obesity among adults in the US, it is necessary to develop comprehensive behavioral prevention programs that integrate educational strategies to reduce screen-based sedentary behavior and tobacco use, especially at higher education institutions. This, in turn, will help in reducing mental health issues like depressive symptoms among college students and promote public health.

It is worth noting that the combined overall effects of physical activity and HRBs on depressive symptoms have not been studied in the past, and this is the first study to examine their relationships by gender and weight status groups, respectively. Hence, these findings may be interpreted with caution due to the cross-sectional design, and future research with a more advanced research design is needed to report the cause–effect relationships. It is important for future studies to determine the type (e.g., computer, TV, social media, etc.) and the extent of screen-based behavior in this population so screen-time guidelines and recommendations can be created effectively and feasibly [25]. More longitudinal research is needed to understand the cause–effect relationship with repeated measures and should use structural equation modeling to determine mediation/moderation effects among the studied associations. From a public health perspective, health promotion strategies should be tailored to weight management, limiting daily screen-based behaviors, and reducing or eliminating e-cigarette use in order to prevent depression among young adults. It is possible that individuals partake in HRBs as a coping mechanism for depression, so understanding the mechanisms of participation in the first place is vital for future interventions to improve the health of young adults.

The unique perspective of this study was to understand the effects of health-risk behaviors on depressive symptoms according to the specific sociodemographics. Even though validated assessments were used to measure activity levels, these assessments were not objectively measured, so the risk of over- or underestimating the time spent on sedentary behaviors or physical activity should be considered. The instrument for measuring depressive symptoms is not a diagnostic tool for screening depression but rather a tool to indicate depressive symptoms, so it would be warranted if future research would include individuals diagnosed with depression to better understand the effects of HRBs on depressive symptoms among this vulnerable population. Also, this sample was recruited from one area in North Texas, so its findings may be hard to generalize, especially when considering lifestyle behaviors.

## 5. Conclusions

This study provides theory-guided evidence regarding the influence of young adults’ HRBs on depressive symptoms, as well as the gender and obesity disparities in the identified associations. This study considered multiple levels of physical activity, sedentary behavior, and tobacco use for a comprehensive overview of various HRB measures and their combined effects on depression among college-aged young adults. The findings highlight specific demographics that are at higher risk for depressive symptoms through various contexts of HRBs. Strategies aimed at relieving depressive symptoms among college-aged young adults can be enhanced by reducing or eliminating screen time and tobacco use among this population. Such strategies should also be tailored to groups with different demographics and weight status.

## Figures and Tables

**Table 1:** Demographic statistics of the study participants (*n* = 366).

Variables	N (%)	Mean (SD)
Sex		
Male	146 (39.9)	
Female	220 (60.1)	
Race		
White	113 (30.9)	
Black	59 (16.1)	
Hispanic	85 (23.2)	
Asian	37 (10.1)	
Other	72 (19.7)	
Education		
Freshman	25 (6.8)	
Sophomore	29 (7.9)	
Junior	83 (22.7)	
Senior	164 (44.8)	
Graduate	65 (17.8)	
Weight Status		
Underweight (BMI < 18.5)	5 (1.4)	
Health weight (18.5 ≤ BMI ≤ 25)	190 (51.9)	
Overweight (25 ≤ BMI ≤ 30)	106 (29)	
Obese (BMI ≥ 30)	65 (17.8)	
BMI		25.55 (4.98)
Age		22.59 (3.54)

BMI = body mass index.

**Table 2:** Correlation analysis among study variables.

Variables	1	2	3	4	5	6	7
Depressive Symptoms	-						
MVPA	−0.12 *	-					
LPA	−0.02	0.36 **	-				
Screen-based SB (h)	0.17 **	−0.12 *	−0.05	-			
Non-screen-based SB (h)	0.08	−0.06	0.01	0.46 **	-		
Cigarette	0.03	−0.06	0.03	0.05	0.10	-	
E-cigarette	0.14 **	0.08	0.11 **	0.02	0.02	0.23 *	-
Mean	16.84	45.58	11.04	9.47	5.70	0.09	0.19
SD	10.31	23.91	7.47	4.03	3.95	0.45	0.61

MVPA = moderate-to-vigorous physical activity; LPA = light physical activity; SB = sedentary behavior; * = *p* < 0.05; ** *p* < 0.01.

**Table 3:** Hierarchical regression analysis results for males and females (*n* = 366).

Variables	Steps	Male (*n* = 146)				Female (*n* = 220)			
		R^2^	β	*t*	*p*	R^2^	β	*t*	*p*
	Step 1	0.04				0.01			
	Step 2	0.12 *				0.07 *			
BMI			0.13	1.55	0.12		0.06	0.85	0.39
MVPA			−0.13	−1.53	0.13		−0.05	−0.75	0.45
LPA			−0.06	−0.63	0.53		0.08	1.12	0.26
Screen-based SB			0.15	1.53	0.13		0.14	1.93	0.05
Non-screen-based SB			−0.16	−1.71	0.09		0.10	1.28	0.20
Cigarette			−0.13	−1.53	0.13		0.03	0.50	0.62
E-cigarette			0.23	2.64	0.01		0.11	1.61	0.11

BMI = body mass index; MVPA = moderate-to-vigorous physical activity; LPA = light physical activity; SB = sedentary behavior; * *p* < 0.05.

**Table 4:** Hierarchical regression analysis results for the healthy weight group and overweight/obese group (*n* = 366).

Variables	Steps	Healthy Weight (*n* = 190)				Overweight/Obese (*n* = 171)			
		R^2^	β	*t*	*p*	R^2^	β	*t*	*p*
	Step 1	0.03				0.05 *			
	Step 2	0.03				0.14 *			
Sex			0.10	1.27	0.21		0.14	1.84	0.07
MVPA			−0.09	−1.05	0.29		−0.10	−1.2	0.23
LPA			0.05	0.62	0.53		−0.07	−0.81	0.42
Screen-based SB			0.09	0.97	0.33		0.19	2.36	0.02
Non-screen-based SB			0.05	0.97	0.33		−0.03	0.41	0.68
Cigarette			−0.07	−0.88	0.38		0.01	0.15	0.88
E-cigarette			0.08	1.04	0.30		0.23	3.09	<0.01

BMI = body mass index; MVPA = moderate-to-vigorous physical activity; LPA = light physical activity; SB = sedentary behavior; * *p* < 0.05.

**Table 5:** Gender and weight status differences among study variables (*n* = 366).

Variables	Male (*n* = 146)	Female (*n* = 215)	Healthy Weight (*n* = 190)	Overweight/Obese (*n* = 171)
Depressive Symptoms	14.81 (9.60)	18.23 (10.60)	16.53 (10.29)	17.17 (10.39)
MVPA	52.83 (24.55)	41.09 (22.33)	43.39 (24.16)	38.14 (19.44)
LPA	11.51 (8.35)	10.72 (6.85)	11.59 (7.21)	9.70 (6.24)
Screen-based SB (h)	8.84 (4.11)	9.92 (3.95)	9.35 (4.24)	9.64 (3.82)
Non-screen-based SB (h)	5.80 (3.92)	5.68 (3.99)	5.28 (3.45)	6.22 (4.40)
Cigarette	0.09 (0.44)	0.09 (0.45)	0.05 (0.45)	0.13 (0.56)
E-cigarette	0.19 (0.65)	0.18 (0/58)	0.18 (0.61)	0.19 (0.62)

MVPA = moderate-to-vigorous physical activity; LPA = light physical activity; SB = sedentary behavior.

## Data Availability

Data may be available upon request.

## References

[1] National Institute of Mental Health Major Depression. https://www.nimh.nih.gov/health/statistics/major-depression.

[2] Deroma V.M., Leach J.B., Leverett J.P. (2009). The relationship between depression and college academic performance. Coll. Stud. J..

[3] Cuijpers P., Vogelzangs N., Twisk J., Kleiboer A., Li J., Penninx B.W. (2014). Comprehensive meta-analysis of excess mortality in depression in the general community versus patients with specific illnesses. Am. J. Psychiatry.

[4] Vos T., Abajobir A.A., Abate K.H., Abbafati C., Abbas K.M., Abd-Allah F., Abdulkader R.S., Abdulle A.M., Abebo T.A., Abera S.F. (2017). Global, regional, and national incidence, prevalence, and years lived with disability for 328 diseases and injuries for 195 countries, 1990–2016: A systematic analysis for the Global Burden of Disease Study 2016. Lancet.

[5] Buchanan J.L. (2012). Prevention of depression in the college student population: A review of the literature. Arch. Psychiatr. Nurs..

[6] Bayram N., Bilgel N. (2008). The prevalence and socio-demographic correlations of depression, anxiety and stress among a group of university students. Soc. Psychiatry Psychiatr. Epidemiol..

[7] Wiium N., Breivik K., Wold B. (2015). Growth Trajectories of Health Behaviors from Adolescence through Young Adulthood. Int. J. Environ. Res. Public Health.

[8] Centers for Disease Control and Prevention Health Risk Behaviors Measure Definitions. https://www.cdc.gov/places/measure-definitions/unhealthy-behaviors/index.html#smoking.

[9] Villanti A.C., Niaura R.S., Abrams D.B., Mermelstein R. (2019). Preventing Smoking Progression in Young Adults: The Concept of Prevescalation. Prev. Sci..

[10] Corder K., Winpenny E., Love R., Brown H.E., White M., Sluijs E.V. (2019). Change in physical activity from adolescence to early adulthood: A systematic review and meta-analysis of longitudinal cohort studies. Br. J. Sports Med..

[11] Upadhyay J., Farr O., Perakakis N., Ghaly W., Mantzoros C. (2018). Obesity as a disease. Med. Clin..

[12] Allinson J.P., Hardy R., Donaldson G.C., Shaheen S.O., Kuh D., Wedzicha J.A. (2017). Combined impact of smoking and early-life exposures on adult lung function trajectories. Am. J. Respir. Crit. Care Med..

[13] Hart P.D., Benavidez G., Erickson J. (2017). Meeting Recommended Levels of Physical Activity in Relation to Preventive Health Behavior and Health Status Among Adults. J. Prev. Med. Public Health.

[14] Jamal A., Phillips E., Gentzke A.S., Homa D.M., Babb S.D., King B.A., Neff L.J. (2018). Current Cigarette Smoking Among Adults—United States, 2016. MMWR Morb. Mortal. Wkly. Rep..

[15] Piercy K.L., Troiano R.P., Ballard R.M., Carlson S.A., Fulton J.E., Galuska D.A., George S.M., Olson R.D. (2018). The physical activity guidelines for Americans. JAMA J. Am. Med. Assoc..

[16] Owen N., Sparling P.B., Healy G.N., Dunstan D.W., Matthews C.E. (2010). Sedentary behavior: Emerging evidence for a new health risk. Mayo Clin. Proc..

[17] Panahi S., Tremblay A. (2018). Sedentariness and health: Is sedentary behavior more than just physical inactivity?. Front. Public Health.

[18] Castro O., Bennie J., Vergeer I., Bosselut G., Biddle S.J.H. (2020). How Sedentary Are University Students? A Systematic Review and Meta-Analysis. Prev. Sci..

[19] Vella C.A., Taylor K., Nelson M.C. (2020). Associations of leisure screen time with cardiometabolic biomarkers in college-aged adults. J. Behav. Med..

[20] Lubans D., Richards J., Hillman C., Faulkner G., Beauchamp M., Nilsson M., Kelly P., Smith J., Raine L., Biddle S. (2016). Physical activity for cognitive and mental health in youth: A systematic review of mechanisms. Pediatrics.

[21] Feng Q., Zhang Q.-L., Du Y., Ye Y.-L., He Q.-Q. (2014). Associations of Physical Activity, Screen Time with Depression, Anxiety and Sleep Quality among Chinese College Freshmen. PLoS ONE.

[22] Wu X., Tao S., Zhang Y., Zhang S., Tao F. (2015). Low Physical Activity and High Screen Time Can Increase the Risks of Mental Health Problems and Poor Sleep Quality among Chinese College Students. PLoS ONE.

[23] Zhou H., Dai X., Lou L., Zhou C., Zhang W. (2021). Association of sedentary behavior and physical activity with depression in sport university students. Int. J. Environ. Res. Public Health.

[24] Zhang X., Gu X., Zhang T., Keller J.M. (2020). The mediating roles of sleep quality and sedentary behavior between physical fitness and depression among female college freshmen. J. Am. Coll. Health.

[25] Wang X., Li Y., Fan H. (2019). The associations between screen time-based sedentary behavior and depression: A systematic review and meta-analysis. BMC Public Health.

[26] Spindle T.R., Hiler M.M., Cooke M.E., Eissenberg T., Kendler K.S., Dick D.M. (2017). Electronic cigarette use and uptake of cigarette smoking: A longitudinal examination of U.S. college students. Addict. Behav..

[27] North C., Marti C.N., Loukas A. (2021). Longitudinal impact of depressive symptoms and peer tobacco use on the number of tobacco products used by young adults. Int. J. Environ. Res. Public Health.

[28] Marsden D.G., Loukas A., Chen B., Perry C.L., Wilkinson A.V. (2019). Associations between frequency of cigarette and alternative tobacco product use and depressive symptoms: A longitudinal study of young adults. Addict. Behav..

[29] King J.L., Reboussin B.A., Spangler J., Ross J.C., Sutfin E.L. (2018). Tobacco product use and mental health status among young adults. Addict. Behav..

[30] Girgus J.S., Yang K. (2015). Gender and depression. Curr. Opin. Psychol..

[31] Marmorstein N.R., Iacono W.G., Legrand L. (2014). Obesity and depression in adolescence and beyond: Reciprocal risks. Int. J. Obes..

[32] Radloff L.S. (1977). The CES-D Scale: A Self-Report Depression Scale for Research in the General Population. Appl. Psychol. Meas..

[33] Godin G. (2011). The Godin-Shephard leisure-time physical activity questionnaire. Health Fit. J. Can..

[34] Godin G., Shephard R. (1997). Godin leisure-time exercise questionnaire. Med. Sci. Sports Exerc..

[35] Shaikh H.M., Patterson M.S., Lanning B., Umstattd Meyer M.R., Patterson C.A. (2018). Assessing college students’ use of campus recreation facilities through individual and environmental factors. Recreat. Sports J..

[36] Rosenberg D.E., Norman G.J., Wagner N., Patrick K., Calfas K.J., Sallis J.F. (2010). Reliability and validity of the Sedentary Behavior Questionnaire (SBQ) for adults. J. Phys. Act. Health.

[37] Little R.J. (1988). A test of missing completely at random for multivariate data with missing values. J. Am. Stat. Assoc..

[38] Portney L.G., Watkins M.P. (2009). Foundations of Clinical Research: Applications to Practice.

[39] Zhang D., Gabriel K.P., Sidney S., Sternfeld B., Jacobs D., Whitaker K.M. (2021). Longitudinal bidirectional associations of physical activity and depressive symptoms: The CARDIA study. Prev. Med. Rep..

[40] Nakagawa T., Koan I., Chen C., Matsubara T., Hagiwara K., Lei H., Hirotsu M., Yamagata H., Nakagawa S. (2020). Regular Moderate- to Vigorous-Intensity Physical Activity Rather Than Walking Is Associated with Enhanced Cognitive Functions and Mental Health in Young Adults. Int. J. Environ. Res. Public Health.

[41] Blough J., Loprinzi P.D. (2018). Experimentally investigating the joint effects of physical activity and sedentary behavior on depression and anxiety: A randomized controlled trial. J. Affect. Disord..

[42] Obisesan O.H., Mirbolouk M., Osei A.D., Orimoloye O.A., Uddin S.M.I., Dzaye O., El Shahawy O., Al Rifai M., Bhatnagar A., Stokes A. (2019). Association Between e-Cigarette Use and Depression in the Behavioral Risk Factor Surveillance System, 2016–2017. JAMA Netw. Open.

[43] Leventhal A.M., Zvolensky M.J. (2015). Anxiety, depression, and cigarette smoking: A transdiagnostic vulnerability framework to understanding emotion–smoking comorbidity. Psychol. Bull..

[44] Lechner W.V., Janssen T., Kahler C.W., Audrain-McGovern J., Leventhal A.M. (2017). Bi-directional associations of electronic and combustible cigarette use onset patterns with depressive symptoms in adolescents. Prev. Med..

[45] Ramo D.E., Young-Wolff K.C., Prochaska J.J. (2015). Prevalence and correlates of electronic-cigarette use in young adults: Findings from three studies over five years. Addict. Behav..

[46] Becker T.D., Arnold M.K., Ro V., Martin L., Rice T.R. (2021). Systematic review of electronic cigarette use (vaping) and mental health comorbidity among adolescents and young adults. Nicotine Tob. Res..

[47] Lanza H.I., Pittman P., Batshoun J. (2017). Obesity and Cigarette Smoking: Extending the Link to E-cigarette/Vaping Use. Am. J. Health Behav..

[48] Cena H., Porri D., De Giuseppe R., Kalmpourtzidou A., Salvatore F.P., El Ghoch M., Itani L., Kreidieh D., Brytek-Matera A., Kolčić I. (2021). How healthy are health-related behaviors in university students: The HOLISTic study. Nutrients.

[49] Stierman B., Afful J., Carroll M.D., Chen T.-C., Davy O., Fink S., Fryar C.D., Gu Q., Hales C.M., Hughes J.P. (2021). National Health and Nutrition Examination Survey 2017–March 2020 prepandemic data files development of files and prevalence estimates for selected health outcomes. National Health Statistics Reports.

